# Generative Artificial Intelligence–Driven Audio Learning Materials for Clinician-Educators: An Exploratory Feasibility Study of Radio- and Narrative-Style Podcasts Using NotebookLM

**DOI:** 10.31662/jmaj.2026-0019

**Published:** 2026-08-14

**Authors:** Hiroki Yasuhara, Hajime Kasai, Osamu Nomura, Ikuo Shimizu

**Affiliations:** 1 Division of Primary Health Care, Faculty of Medical Sciences, University of Fukui, Yoshida-gun, Fukui, Japan; 2 Department of Medical Education, Graduate School of Medicine, Chiba University, Chiba, Japan; 3 Health Professional Development Center, Chiba University Hospital, Chiba, Japan; 4 Department of Respirology, Graduate School of Medicine, Chiba University, Chiba, Japan; 5 Medical Education Development Center, Gifu University, Gifu, Japan

**Keywords:** generative artificial intelligence, large language model, health professions education, podcasts as topic

## Abstract

**Introduction:**

Clinician-educators must keep pace with rapidly evolving evidence while improving their teaching skills. However, time constraints often limit their participation in traditional faculty development, thus increasing the demand for portable medical education resources. To address this gap, we conducted an exploratory feasibility study to develop and evaluate a source-grounded approach for producing Japanese audio learning materials using generative artificial intelligence (AI).

**Methods:**

Peer-reviewed health professions education papers in portable document format (PDF) served as primary sources. Gemini 2.5 Pro generated two supplemental script formats: fictional listener letters describing teaching dilemmas for a radio dialogue and metaphorical fables for a narrative story. We uploaded the scripts and PDF documents to NotebookLM and prompted the AI hosts to respond while adhering to the sources. Audio Overviews were generated in Japanese (short setting). A convenience sample of six practicing physicians—three medical education specialists and three clinician-educators—independently evaluated both formats using a modified Revised Medical Education Translational Resources: Impact and Quality scale (0-3 per item). Reviewers also provided open-ended feedback.

**Results:**

Structure and production (Item 3) and clinical utility (Item 6) consistently received high ratings across evaluators for both formats (scores: 2-3). Clinician-educators found the podcasts extremely helpful for those teaching between busy clinical duties, whereas specialists cautioned against potential oversimplification. Transparency of the editorial and prepublication review processes (Item 5) consistently received the lowest scores (0-1). Evaluators found the synthesized voices natural but noted technical limitations, primarily occasional mispronunciations of specialized Japanese terms. They recommended including clear transparency statements outside the podcast directing learners to the original PDF documents.

**Conclusions:**

Preliminary evidence suggests that this approach can rapidly generate structured, source-grounded medical education podcasts for busy clinician-educators. However, human curation at multiple stages, transparent reporting, and learner-based evaluation are required to ensure accuracy, trustworthiness, and educational impact.

## Introduction

Clinician-educators must constantly update their knowledge to provide high-quality care; yet, they face significant time constraints due to their dual responsibilities. These constraints often prevent them from engaging in traditional group-based programs or time-consuming passive learning ^(1)^. Conventional educational resources require fixed schedules or physical attendance, which do not fit the busy schedules of clinician-educators. The timing and format of faculty development offerings are often in conflict with ongoing clinical responsibilities, requiring faculty to sacrifice personal time outside of work hours or use limited continuing medical education time, which can ultimately lead to low program attendance ^(2)^. In this context, podcasts serve as a flexible, “just-in-time” educational medium ^(3)^. This format allows clinicians to acquire new information while engaged in other activities, making it valuable for its portability and efficiency ^(3)^. Despite their utility, creating high-quality, engaging audio content remains a resource-intensive challenge.

While podcasts are effective for clinical updates, resources dedicated to faculty development, specifically those covering foundational educational literature, remain scarce because of the production burden. The integration of generative artificial intelligence (GenAI) offers a novel solution to this problem. GenAI provides substantial opportunities to transform various facets of medical education, most notably by simplifying the development of educational materials ^(4)^. GenAI can streamline the creation of high-quality educational content by automating the restructuring of text-based information into auditory formats. To leverage this potential while ensuring accuracy, we drew upon a recent framework that integrates tools such as Google NotebookLM into health professions education ^(5)^. This approach emphasizes a systematic workflow across the “create-convert-collaborate” spectrum, underpinned by integrated curation processes to ensure quality assurance ^(5)^. Consequently, this paper presents an exploratory feasibility study of the “GenAI-MedEd-Podcast” approach, which operationalizes this framework to transform peer-reviewed articles into two distinct audio formats: a radio-style dialogue and a narrative fable. We intentionally limited the source materials to health professions education papers because the primary aim of this study was to explore the feasibility and perceived utility of these materials for supporting faculty development among clinician-educators, rather than to create general educational podcasts for broader professional audiences.

## Materials and Methods

### Development of the podcast

We developed the GenAI-MedEd-Podcast workflow as a two-step process using Google’s GenAI tools, as illustrated in Figure 1. In the first step, we employed Gemini 2.5 Pro (Google LLC, Mountain View, CA, USA), a large language model, to generate supplementary text components derived from peer-reviewed papers on health professions education. Specifically, the model was prompted to create fictional “listener letters” that presented educational dilemmas for a radio-style dialogue and metaphorical “fables” that distilled core principles for a narrative-style format (Supplementary Appendix 1). This step was essential to provide context and narrative structure, as the subsequent tool, NotebookLM, relies on uploaded source materials to generate audio content ^(3)^.

**Figure 1.**
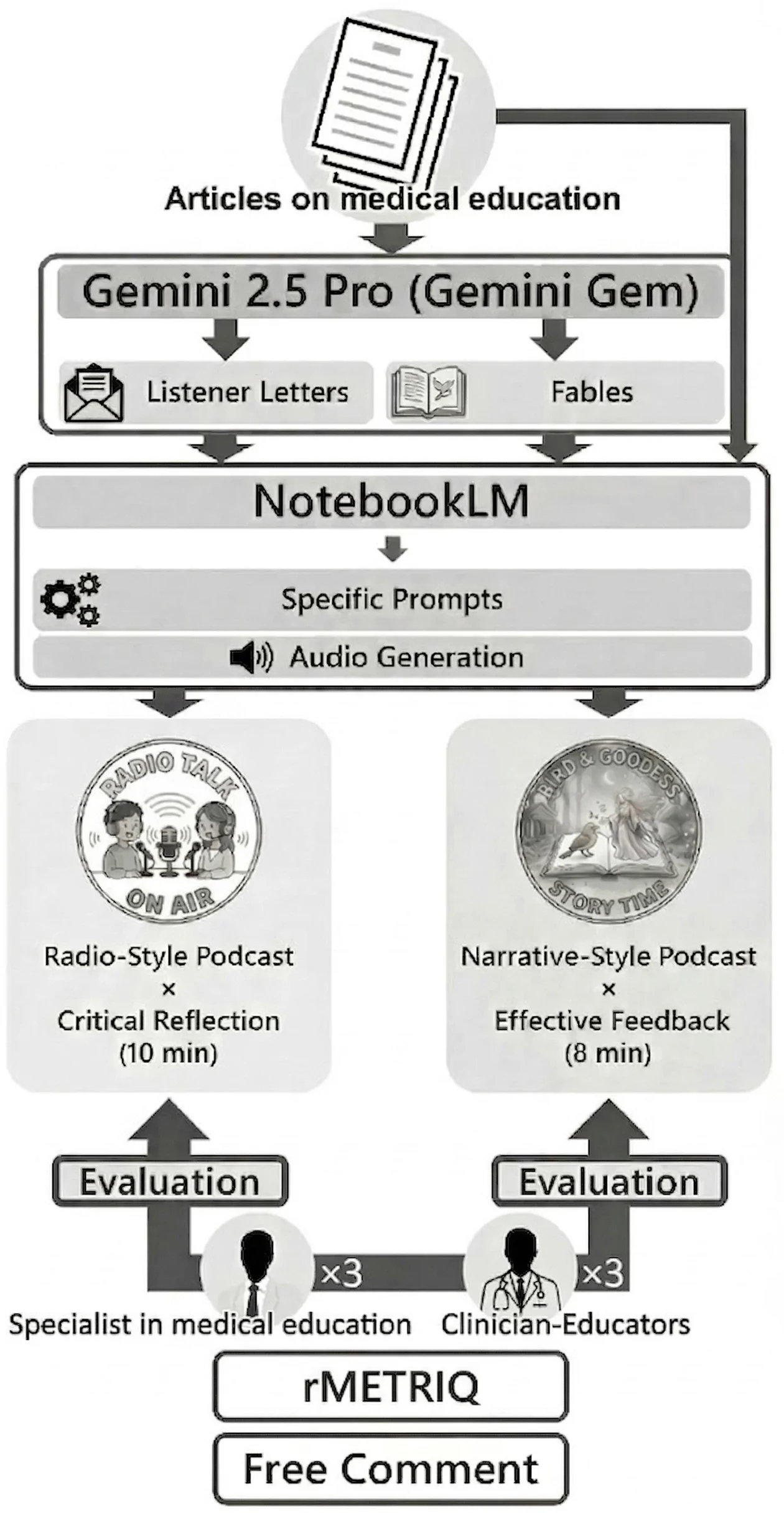
Workflow for the generation and expert evaluation of GenAI-driven pedagogical podcasts. The process consists of two main phases: development and evaluation. In the development phase, Gemini 2.5 Pro generates supplementary scripts (listener letters and fables) from peer-reviewed papers on health professions education. These scripts and the original PDF documents are uploaded to NotebookLM, which generates audio content (radio- and narrative-style podcasts) based on specific prompts. In the evaluation phase, three medical education specialists and three general clinician-educators assess the generated podcasts using a modified rMETRIQ score and provide open-ended qualitative feedback. GenAI: generative artificial intelligence; PDF: portable document format; rMETRIQ: Revised Medical Education Translational Resources: Impact and Quality.

In the second step, these generated texts and the original source portable document format (PDF) documents were uploaded to NotebookLM. Unlike general-purpose chatbots, NotebookLM operates on a source-grounding architecture, ensuring that its outputs are derived strictly from the uploaded materials ^(3)^. We used the tool’s “Audio Overview” feature to synthesize these inputs into a conversational format. Specific prompts directed the AI hosts to integrate the supplementary texts with the source literature (Supplementary Appendix 2). For instance, prompts instructed the hosts to address the listener’s dilemma using evidence-based solutions from the PDF documents or to deconstruct the fable’s metaphors to explain educational theories. To maximize accessibility for the target audience, the GenAI system was prompted to generate the dialogue directly in Japanese, and the “Short” duration setting was selected to suit busy schedules.

### Evaluation

To assess the feasibility and perceived utility of the generated content, we selected two representative articles: a practical guide for teaching reflection (Aronson, Med Teach 2011) for the radio-style dialogue and foundational guidelines for clinical feedback (Ende, JAMA 1983) for the narrative-style story. Using a convenience sampling approach through the authors’ professional networks, we recruited two distinct groups of practicing physicians to evaluate these audio materials: three medical education specialists with master’s degrees in medical education and three general clinician-educators without formal educational training who routinely supervise trainees. This composition ensured that the assessment incorporated the perspectives of both educational researchers (content experts) and busy clinicians (the primary target audience). Between November 2025 and March 2026, all six evaluators independently listened to the generated audio files on their own devices at their convenience and assessed them using a structured instrument. We provide Supplementary Audio 1 (radio-style dialogue) and Supplementary Audio 2 (narrative-style story) as sample audio files created using this approach.

We used a modified version of the Revised Medical Education Translational Resources: Impact and Quality score, an instrument originally validated for assessing online medical education blogs ^(6)^. Given the auditory nature of our materials, we adapted the scale to suit audio learning materials; for instance, Item 3 was revised from “well-written” to “well-produced and clearly structured.” Items irrelevant to prerelease GenAI content, such as disclosure of conflicts of interest (Item 5) and postpublication comments (Item 7), were excluded. To capture the specific educational value of the podcasts, we added two new items: “utility for clinical practice or learning” and “interest and engagement.” Each item was scored on a scale of 0 to 3 (Table 1), and the evaluators also provided open-ended qualitative feedback regarding the potential utility and challenges of the content.

**Table 1.** Modified rMETRIQ Score Descriptors for GenAI-Generated Podcast Evaluation.

	Evaluation item	Score: 0	Score: 1	Score: 2	Score: 3
1	Does the podcast provide enough background information to situate the user?	No background information; difficult to understand	Some background information but insufficient or unclear	Sufficient background information provided to aid understanding	Rich, detailed background information that fully supports understanding
2	Does the podcast contain an appropriate amount of information for its length?	Insufficient information	Too much or unnecessary information; inappropriate amount	Mostly appropriate amount of information; key information included	Perfectly balanced amount of information; concise yet complete
3	Is the podcast well-produced and clearly structured?	Poor audio quality, very hard to understand	Some audio issues or unclear structure	Good audio quality, mostly clear structure	Excellent audio quality, logical, and well-structured
4	Does the podcast cite its references?	No references provided	Few or inappropriate references	Appropriate references cited	Ample and appropriate references clearly provided
5	Are the editorial and prepublication peer review processes clearly outlined?	No information on editorial/review process	Some information provided but incomplete	Mostly clear about process	Detailed and clearly described process
6	How useful is the content for clinical practice or learning?	Not useful; no valuable learning points	Slightly useful; contains a few minor learning points	Useful; contains several valuable learning points	Highly useful; contains key learning points that are essential for learners
7	How interesting and engaging is the content?	Not interesting or engaging	Somewhat interesting but not particularly engaging	Interesting and engaging for the listener	Highly interesting and engaging; likely to inspire further learning
Free-text comments					

GenAI: generative artificial intelligence; rMETRIQ: Revised Medical Education Translational Resources: Impact and Quality.

## Results

The individual rater scores, medians, and ranges for both podcast formats, evaluated by three medical education specialists and three clinician-educators, are presented in Table 2. Across all six evaluators, Item 3 (structure/production) and Item 6 (utility) consistently received high scores (ranging from 2 to 3) for both formats. Conversely, Item 5 (editorial/review process) consistently received the lowest scores (ranging from 0 to 1) across both groups and formats.

**Table 2.** Quantitative evaluation of GenAI-generated podcasts using a modified rMETRIQ score (n = 6: three medical education specialists and three general clinician-educators).

Items and formats		S1	S2	S3	Median (range)	C1	C2	C3	Median (range)
Background									
	Radio	2	2	2	2.0 (2–2)	3	3	3	3.0 (3–3)
	Narrative	3	2	2	2.0 (2–3)	3	2	2	2.0 (2–3)
Appropriate amount of information									
	Radio	3	3	2	3.0 (2–3)	3	3	3	3.0 (3–3)
	Narrative	2	2	3	2.0 (2–3)	3	3	2	3.0 (2–3)
Well-produced									
	Radio	3	3	3	3.0 (3–3)	2	3	2	2.0 (2–3)
	Narrative	2	3	3	3.0 (2–3)	2	3	3	3.0 (2–3)
Cites references									
	Radio	3	3	2	3.0 (2–3)	3	3	2	3.0 (2–3)
	Narrative	3	2	3	3.0 (2–3)	2	3	3	3.0 (2–3)
Editorial process									
	Radio	1	1	0	1.0 (0–1)	1	1	3	1.0 (1–3)
	Narrative	1	1	1	1.0 (1–1)	1	1	0	1.0 (0–1)
Utility									
	Radio	3	2	3	3.0 (2–3)	3	3	3	3.0 (3–3)
	Narrative	2	2	3	2.0 (2–3)	3	3	3	3.0 (3–3)
Interesting/engaging									
	Radio	2	3	2	2.0 (2–3)	3	2	3	3.0 (2–3)
	Narrative	3	3	2	3.0 (2–3)	3	2	2	2.0 (2–3)

C: general clinician-educator; GenAI: generative artificial intelligence; rMETRIQ: Revised Medical Education Translational Resources: Impact and Quality; S: medical education specialist.

Qualitative feedback provided context for these quantitative scores, particularly regarding clinical utility (Item 6). Clinician-educators awarded the maximum score (3) for both formats, noting that the podcasts were “extremely helpful for those teaching between busy clinical duties” and “lowered the barrier to initiating self-directed learning.” While medical education specialists also recognized the utility, they assigned slightly more varied scores (2 to 3), cautioning that the content “might feel somewhat oversimplified for listeners familiar with healthcare education.”

Regarding production quality (Item 3), both groups of evaluators praised the synthesized voices, describing them as “much easier to listen to than expected for AI audio” with “natural conversational exchanges.” However, they identified specific technical limitations. These included occasional mispronunciations of specialized Japanese terminology such as *seisatsu* (reflection).

The consistently low scores for the editorial process (Item 5) reflected a lack of transparency within the audio itself. Both groups noted that the peer-review process “was not mentioned within the podcast.” However, clinician-educators offered a practical solution, suggesting that providing this information “in the description section outside the podcast would be sufficient.”

## Discussion

This exploratory feasibility study provides preliminary evidence regarding the feasibility and perceived utility of using the GenAI-MedEd-Podcast approach to develop audio materials for busy clinician-educators. The strong consensus on production quality (Item 3) and clinical utility (Item 6) among clinician-educators suggests that NotebookLM can effectively translate complex educational theories into accessible auditory formats, lowering the barrier to self-directed learning. However, medical education specialists cautioned that the generated content might feel oversimplified. This concern aligns with recent evidence demonstrating that large language models exhibit a “generalization bias” when summarizing scientific research, frequently omitting crucial nuances, limitations, and qualifiers present in the original texts, leading to unwarranted overgeneralizations ^(7)^. Furthermore, evaluators identified technical limitations, primarily occasional mispronunciations of specialized Japanese terminology, and consistently assigned low scores to transparency of the editorial process (Item 5). These findings highlight a critical challenge: while GenAI can restructure information into engaging formats, it does not inherently ensure pedagogical precision or provide transparency regarding its curation process.

To address these challenges, our approach aligns with a framework that integrates the “create-convert-collaborate” spectrum with “curate” processes ^(5)^. This method posits that AI tools should function not as autonomous creators but as engines requiring rigorous oversight through systematic curation ^(5)^. Furthermore, the safe deployment of AI systems in the medical domain demands a “human-in-the-loop” baseline, where qualified professionals critically evaluate and validate all outputs ^(8)^. Consequently, human curation must occur explicitly at three stages within our workflow. First, after text generation, educators must verify that the supplementary scripts accurately reflect the source literature without hallucination. Second, after audio generation, they must review the output to correct any mispronunciations of specialized terminology and ensure that the AI tool has not introduced unwarranted overgeneralizations. Third, before distribution, the final audio must undergo peer review by another medical education specialist to confirm pedagogical validity. Finally, to ensure transparency, the audio introduction or accompanying text must clearly state that the content is AI-generated and direct learners to consult the original peer-reviewed articles.

Beyond immediate practical utility, the potential impact and future trajectory of this approach align with emerging global trends in the digital transformation of AI-assisted medical education. According to the substitution augmentation modification redefinition (SAMR) model ^(9)^, the integration of information technology into education is conceptualized as a four-level taxonomy: substitution, augmentation, modification, and redefinition. At the substitution level, the proposed method converts text-based papers into an auditory format without functionally changing the core task of information consumption. For augmentation, it uses functional improvements inherent to the podcast medium. Specifically, conversational dialogue and “edutainment” value enhance learner engagement more effectively than traditional monologue lectures ^(2)^. The process reaches the modification level by redesigning explanations into a structured “talk/theme” format optimized for learning “how to teach” through audio. Looking forward, the methodology has the potential for redefinition, as NotebookLM now incorporates capabilities to generate visual aids such as infographics and slides. By integrating these multimodal outputs, the method could evolve into an adaptive learning environment that accommodates diverse learning styles simultaneously, a development that reflects the broader transformative opportunities GenAI offers for efficient and scalable educational content creation ^(4)^. This progression toward a comprehensive multimodal learning experience offers a practical model for busy clinician-educators to efficiently acquire educational expertise. Although this study focused on health professions education to address the specific needs of clinician-educators, the underlying methodology is not inherently limited to this domain. The process of converting complex textual information into structured, conversational, and narrative-based audio content has potential applicability across a wide range of clinical topics, including clinical guidelines, disease education, and continuing professional development. Furthermore, this approach may be extended beyond medicine to other knowledge-intensive disciplines where learners face similar challenges of information overload and time constraints. Thus, this study can be positioned as an initial application within medical education, with broader implications for scalable and personalized learning across domains.

This study has several limitations inherent to its exploratory feasibility design. First, the evaluation relied on a small convenience sample of only three medical education specialists and three clinician-educators. This limited number of specialists may not provide a sufficiently comprehensive review of the pedagogical content, and the small group of clinician-educators may not fully represent the broader population of the target audience. Consequently, perceptions of GenAI-generated content may vary across different professional contexts and individual learning preferences. Second, the evaluation was based entirely on subjective self-reported measures (perceived utility and quality) rather than objective outcomes. Finally, this study did not assess actual learner outcomes, behavioral changes in teaching practices, or the implementation of these materials in authentic educational settings. Future research should employ more rigorous methodologies, including larger, representative samples and the measurement of objective educational outcomes, to establish the true effectiveness of this approach.

In conclusion, preliminary evidence from this exploratory study suggests that this approach provides a feasible method for developing GenAI-generated podcasts that may support the self-directed learning of educational expertise among busy clinician-educators. Although NotebookLM demonstrates a strong capability to structure and vocalize pedagogical content, human curation remains essential to ensure transparency and accuracy. Positioned at the augmentation and modification levels of the SAMR model, this approach has the potential to redefine how clinician-educators acquire and refine teaching expertise. Building upon these initial findings, the next essential step is a systematic, learner-based evaluation of its impact on teaching practices in real-world clinical education settings to determine its true educational effectiveness.

## Article Information

### Acknowledgments

During the conduct of this study, the authors used Google Gemini 2.5 Pro, Gemini 3.0, and NotebookLM. These tools were used for two primary purposes: (1) generating the radio- and narrative-style audio content that served as the study material and (2) providing writing assistance, including English translation and paraphrasing. After using these tools, the authors reviewed and edited the content as necessary and take full responsibility for the content of the publication.

The authors thank Editage (www.editage.jp) for English language editing.

### Author Contributions

Hiroki Yasuhara: conceptualization, methodology, data curation, writing – original draft, and writing – review and editing; Hajime Kasai: conceptualization, methodology, supervision, writing – review and editing, and investigation (content evaluation); Osamu Nomura: supervision, writing – review and editing, and investigation (content evaluation); Ikuo Shimizu: supervision, writing – review and editing, and investigation (content evaluation). All authors have read and approved the published version of the manuscript.

### Conflicts of Interest

None

### Ethics Approval

Not applicable. This study involved the development and evaluation of educational materials by the authors using open data and did not involve human subjects research requiring institutional review board approval.

## Supplement

Supplementary Appendix 1

Supplementary Audio 1 (radio-style dialogue, in Japanese)

Supplementary Audio 2 (narrative-style story, in Japanese)
