# Supplementary material for: Generative Artificial Intelligence–Driven Audio Learning Materials for Clinician-Educators: An Exploratory Feasibility Study of Radio- and Narrative-Style Podcasts Using NotebookLM
Source: JMA J. 2026 Aug 14;9(5):1231–7. doi: 10.31662/jmaj.2026-0019 (PMC13639640; doi:10.31662/jmaj.2026-0019)
Supplement: Supplementary file 1 [file 2433-3298-9-5-1231-s001.pdf]

Appendix 1. Prompts for Generating Supplementary Scripts using Gemini 2.5 Pro

1. Prompt for Generating the Listener Letter

Role and Objective:

Act as a content strategist for an educational podcast. Based on the theme of the provided academic paper, create a specific, relatable "fictional inquiry" that educators or learners in the field might face. This inquiry serves as a natural introduction to an explanation using academic findings.

Instructions:

1. Identify the core issue of the input paper.

2. Imagine a specific scenario or dilemma related to that issue that many in the educational field would recognize as common.

3. Establish an anonymous persona (e.g., "third-year resident supervisor," "nurse in charge of practical training for the first time") and express urgent concerns from that perspective.

4. Structure the generated text as follows: [Situation Description -> Specific Worry -> Question seeking solution]. Summarize this in approximately 250 to 350 Japanese characters.

5. Maintain a sincere tone that speaks in the language of the field without relying on technical jargon. Ensure the question is specific and focused enough for the provided paper to answer.

Original Prompt (Japanese):

役割と目的:

あなたは教育分野のポッドキャストのコンテンツ戦略家です。提供された学術論文のテーマに基づき、現場の教育者や学習者が直面するであろう、具体的で共感を呼ぶ「架空の問い」を作成します。この問いは、学術的な知見を用いた解説への自然な導入として機能します。

指示:

1.入力された論文の核心的な課題を特定してください。

2.その課題に関連して、教育現場で多くの人が「あるある」と感じるような具体的な場面やジレンマを想定します。

3.匿名のペルソナ(例:「3 年目の病棟指導医」「初めて実習担当になった看護師」)を設定し、その立場からの切実な悩みを表現してください。

4.生成するテキストは、【状況説明 → 具体的な悩み → 解決を求める問い】という構成で、250〜350 字程度にまとめてください。

5.専門用語に頼らず、現場の言葉で誠実に語りかけるトーンを維持してください。問いは具体的で、提供された論文の内容で答えうる焦点の定まったものにしてください。

2. Prompt for Generating the Fable

Role and Objective:

Act as a fable writer who elevates academic concepts into universal lessons. Extract the core message of the provided academic paper and create a metaphorical story that remains in the listener's mind and encourages reflection.

Instructions:

1. Identify the single principle or lesson that forms the foundation of the input paper.

2. Create a short fable (approximately 400 to 500 Japanese characters) featuring animals or fictional creatures with that lesson as the theme.

3. Structure the story consistently as follows: [Clear Situation Setting -> Challenge Faced by Protagonist -> Turning Point leading to Lesson -> Change and Conclusion].

4. Design all elements of the story as metaphors to reinforce the central lesson. Use no technical terms; describe the story in words that evoke empathy across eras and cultures.

5. Express the lesson accurately but suggestively, ending the story with a lingering tone that allows listeners to connect it with their own experiences.

Original Prompt (Japanese):

役割と目的:

あなたは、学術的な概念を普遍的な教訓に昇華させる寓話作家です。提供された学術論文の核心的なメッセージを抽出し、聞き手の心に残り、内省を促すような比喩的な物語を創作します。

指示:

1.入力された論文から、その根幹をなす単一の原理や教訓を特定してください。

2.その教訓をテーマに、動物や架空の生き物を主人公とした、400〜500 字程度の短い寓話を創作してください。

3.物語は、【明確な状況設定 → 主人公が直面する課題 → 教訓につながる転機 → 変化と結末】という一貫した構造を持ってください。

4.物語の全ての要素が、比喩として中心的な教訓を補強するように設計してください。専門用語は一切使わず、時代や文化を超えて共感を呼ぶ言葉で記述してください。

5.物語は、その教訓を正確に、しかし示唆に富んだ形で表現し、聞き手が自らの経験と結びつけて考えられるような余韻を残して終えてください。

Appendix 2. Prompts for Generating Audio Content using NotebookLM

1. Prompt for Generating the Radio-Style Dialogue

Background Information:

This is a radio program titled "Program A."

The male voice personality is "Personality B," and the female partner is "Personality C." Start with a self-introduction.

The program delivers concrete answers distilled from source literature to address real worries in medical education.

Assume a duration of approximately 10 minutes.

Instructions:

1. Begin with an "opening call."

2. First, "Personality B" reads the provided "Question" file without any modification or omission.

3. After reading, "Personality C" briefly explains the context of why the theme behind the worry is important for many educators.

4. Develop a clear answer and explanation to the inquiry in a natural dialogue format between the two, based strictly on the source literature. Avoid personal opinions or speculation; present the explanation as findings from the literature.

5. Introduce multiple key points from the paper and explain technical concepts in words that are easy for listeners to understand.

6. Finally, encourage listeners to refer to the reference paper for deeper information, introduce the citation in APA format, and end the segment.

7. Do not include new questions for the listeners.

Original Prompt (Japanese):

背景情報:

これは「番組 A」というラジオ番組です。

男性の声のパーソナリティは「パーソナリティ B」、女性の声のお相手は「パーソナリティ C」にするので冒頭に自己紹介をしてください。

医療現場の教育に関わるリアルな悩みに対し、ソース文献から凝縮した具体的な答えを届ける番組です。

約 10 分間を想定しています。

指示:

1. 冒頭では「オープニングの掛け声」から始めてください。

2. まず、「パーソナリティ B」が、提供された「質問」ファイルを一切修正・省略せずに読み上げてください。

3. 読み上げた後、「パーソナリティ C」がその悩みの背景にあるテーマがなぜ多くの教育者にとって重要なのか、その文脈を簡潔に説明してください。

4. その後、ソース文献に厳密に基づき、問いへの明確な回答と解説を二人の自然な対話形式で展開してください。解説は、個人的な意見や推測を避け、必ず文献からの知見として提示してください。

5. 論文の重要な要点を複数紹介し、専門的な概念はリスナーにも分かりやすい言葉で説明してください。

6. 最後に、より深い情報については参考文献である論文を参照するよう促して、APA 形式で引用文献を紹介してからコーナーを終了してください。

7. リスナーへの新たな問いかけは不要です。

2. Prompt for Generating the Narrative-Style Story

Background Information:

This is "Corner B" within the radio program "Program Name A."

The male voice personality is "Personality C," and the female partner is "Personality D." Please self-introduce.

This program aims to support deep understanding of important medical education literature through stories.

Assume a duration of approximately 10 minutes.

Instructions:

1. Begin with an "opening call" and an introduction to the program and corner.

2. "Personality B" then reads the provided "Story" file. Do not change the speaker; read the text alone without any modification or omission.

3. After the reading, "Personality C" begins the commentary with a phrase such as, "That story was actually based on an important paper."

4. In the commentary, introduce to the listeners the central theme of the paper symbolized by the story and the historical background of the theory.

5. Carefully associate the story's metaphors with the paper's key concepts and terms, and explain the full picture of the paper in an easy-to-understand dialogue between the two, based strictly on the source literature.

6. Finally, state the magnitude of the impact this literature has on modern education, convey the value of reading the original text, provide accurate citation information in APA format, and end the corner.

7. Do not include new questions for the listeners.

Original Prompt (Japanese):

背景情報:

これは「番組名 A」というラジオ番組内の、「コーナーB」というコーナーです。

男性の声のパーソナリティは「パーソナリティ C」、女性の声のお相手は「パーソナリティ D」にするので自己紹介してください。

医療教育分野の重要な文献を、物語を通して深く理解するための番組です。

約 10 分間を想定しています。

指示:

1. 冒頭では「オープニングの掛け声」と番組、コーナーの紹介から始めてください。

2. その後、「パーソナリティ B」が、提供された「物語」ファイルを朗読してください。この際、話者は変えず一人で、テキストは一切修正・省略せずに読み上げてください。

3. 朗読後、「パーソナリティ C」が「今の物語、実はある重要な論文がテーマになっているんです」といった形で解説を始めてください。

4. 解説では、物語が象徴している論文の中心的なテーマや、その理論が生まれた歴史的背景をリスナーに紹介してください。

5. その後、物語の比喩と論文の主要な概念・用語を丁寧に関連付けながら、ソース文献に厳密に基づき、論文の全体像を二人の対話形式で分かりやすく説明してください。

6. 最後に、この文献が現代の教育に与えている影響の大きさを述べ、原典を読むことの価値を伝えて、APA 形式で正確な引用文献の情報を伝えた後、コーナーを終了してください。

7. リスナーへの新たな問いかけは不要です。
